# Knowledge, Attitudes, and Practices Toward Burn Causes and First Aid Management in Jazan Region, Saudi Arabia: Cross-Sectional Study

**DOI:** 10.2196/80594

**Published:** 2025-12-23

**Authors:** Anas Sayegh, Alyazid Y Awaji, Anas Fathuldeen, Atheer M Alshammakhi, Roaa M Alhazmi, Razan F Ageeli, Ghadi A Shamakhi

**Affiliations:** 1 Department of Surgery Faculty of Medicine Jazan University Jazan Saudi Arabia; 2 Department of Plastic and Reconstructive Surgery King Fahad Central Hospital Jazan Saudi Arabia; 3 Department of Plastic Surgery College of Medicine University of Ha'il Ha'il Saudi Arabia; 4 Faculty of Medicine Jazan University Jazan Saudi Arabia

**Keywords:** burns, first aid, knowledge, practice, population, Saudi Arabia

## Abstract

**Background:**

Burn injuries are a major global health problem, particularly in low- and middle-income countries, accounting for most burn-related deaths and disabilities. In Saudi Arabia, burns remain a frequent cause of morbidity, often resulting from domestic accidents involving hot liquids, open flames, or electricity. The Jazan region, with its dense population and reliance on traditional cooking methods, is considered at higher risk. Understanding public knowledge, attitudes, and practices regarding burns and first aid is essential for guiding preventive strategies and health education efforts.

**Objective:**

This study aimed to assess the level of awareness, knowledge, and practices related to burn causes and first aid management among residents of the Jazan region, Saudi Arabia.

**Methods:**

A cross-sectional online survey was conducted using a validated Arabic questionnaire distributed through social media platforms. The questionnaire covered demographics, knowledge of burn causes and types, first aid management, and preventive practices. Data were analyzed using SPSS version 26.0.

**Results:**

Out of 404 participants aged 18-60 years, 228 (56.4%) demonstrated poor knowledge and awareness regarding burn causes and first aid management. The internet was the most commonly reported source of information (171/236, 72.5%), followed by formal courses (76/236, 32.2%), paper leaflets (75/236, 31.8%), television (67/236, 28.4%), and daily newspapers (23/236, 9.7%). Additionally, 215 (53.2%) participants had previously experienced burns, with hot water (136/215, 63.8%) and fire (105/215, 48.8%) cited as the most common causes. The most frequently affected sites were the hand (111/215, 51.6%), arm (85/215, 39.5%), and thigh (49/215, 22.8%).

**Conclusions:**

The study highlights limited awareness and improper first aid practices among the Jazan population. Targeted community-based educational programs are needed to enhance burn prevention and management knowledge.

## Introduction

Burn injuries are a major global cause of morbidity and mortality, posing a significant public health challenge [1]. The World Health Organization states that burn injuries cause about 195,000 fatalities and disability annually, making them a major public health concern. Among individuals aged 5-29 years, fire is the 15th most common cause of death [2]. It is concerning that over 95% of fire-related burn deaths occur in low- and middle-income countries (LMICs). People aged 70 years and older as well as children younger than 5 years have the highest mortality rates [3]. In addition to the high mortality rates, millions of people live with permanent disability and deformities, often facing social rejection and stigma [4].

Evaluating the reasons for burns and assessing the efficiency of first-aid management practices are crucial steps toward addressing and understanding this issue and developing targeted strategies [5]. Additionally, establishing successful preventive plans requires an awareness of the causes of burns [6]. Burns can result from exposure to radiation, heat, electricity, chemicals, and other causes [7,8]. Determining the exact causes and associated risk factors is necessary to set priorities for preventative actions and to allocate resources effectively. It is imperative to investigate the specific factors in Jazan, Saudi Arabia, that cause burn injuries. This means considering local customs, workplace hazards, and any environmental factors specific to the area [9,10]. In addition to identifying burn causes, assessing and analyzing the circumstances in which burn accidents occur can also provide valuable insights for designing safety interventions. For example, understanding the specific situations where burns most frequently happen, whether in households, workplaces, or recreational settings, can help develop targeted prevention strategies. By addressing these specific contexts, interventions can be developed to improve safety practices and reduce the likelihood of burn incidents [5,11].

First aid management plays a critical role in minimizing the severity and complications of burn injuries. Prompt and proper first aid measures can significantly improve outcomes and reduce the long-term impact of burns. However, the effectiveness of first aid management depends on the knowledge and skills of the individuals providing the first care. Therefore, assessing the adequacy of first aid practices is crucial in identifying areas of improvement and implementing targeted educational interventions [12,13]. Research conducted in other regions has offered valuable insights into burn causes and first aid management practices [14-17]. However, it is important to recognize that the factors contributing to burn injuries and the strategies used for first aid management may vary across different geographical locations. Therefore, a focused assessment of burn causes and first aid management practices in Jazan, Saudi Arabia, is necessary to understand the unique challenges and opportunities for intervention in this specific region. This research aimed to conduct a comprehensive assessment of burn causes and first aid management practices in Jazan, Saudi Arabia. This study analyzed the effects of demographics, burn severity, and location in Jazan, Saudi Arabia, aiming to reveal the trends and address knowledge gaps for health care improvement. Our results will help professionals and policymakers, aiding in reducing burn-related morbidity and mortality rates with associated social and economic burdens.

## Methods

### Study Design and Setting

This community-based cross-sectional study was conducted in the Jazan region, located in the southwestern part of Saudi Arabia. Jazan is characterized by varied geographical and socioeconomic conditions, comprising urban, rural, and coastal communities. This study targeted adult residents (aged 18 years and older) living in different governorates of the region. Participants were invited voluntarily to complete the survey, and eligibility was confirmed by screening for age (18 years and older) and residence within the Jazan region.

### Study Population

Eligible participants for inclusion in the survey were individuals who were 18 years or older and voluntarily agreed to participate, residing in the Jazan region. Those younger than 18 years and not Jazan residents were excluded.

### Sample Size

The Raosoft sample size calculator was needed to determine the sample size for this study, incorporating a 5% margin of error, a 95% CI, a 50% response distribution, and population size data of 1,404,997 sourced from the General Authority for Statistics. A total of 385 participants will be needed to reach a 95% CI and a 5% margin of error. The determination of the sample size aimed to ensure the generalizability and relevance of the findings. A nonprobability convenience sampling technique was adopted to facilitate data collection.

### Data Collection Method

Data were collected electronically via Google Forms using a validated and structured questionnaire. The instrument was developed in English, then translated into Arabic, and administered in Arabic (see Multimedia Appendix 1). The questionnaire was specifically developed to assess the knowledge, attitudes, and practices toward burn causes and first aid management among residents of the Jazan region. It consisted of 5 major sections: (1) demographic information (age, sex, education, occupation, income, and residence), (2) previous exposure to burn prevention education or training, (3) knowledge of first aid procedures for burns of different degrees, (4) practical behaviors and responses during burn incidents, and (5) attitudes toward prevention, home safety tools, and awareness methods.

We defined burn first-aid practices and burn depth according to World Health Organization (WHO) criteria [18]. The questionnaire was developed by the research team following an extensive literature review and consultation with 3 public health experts who evaluated the tool for clarity, cultural appropriateness, and content validity. Based on their feedback, necessary modifications were made, and a pilot study involving 20 participants was conducted to assess reliability, yielding a Cronbach α coefficient of 0.75.

### Recruitment

Participants were recruited through a nonprobability convenience sampling approach. The survey link was distributed via major social media platforms (WhatsApp, X, Facebook, Telegram, and Snapchat) and community groups across the Jazan region. Participation was entirely voluntary and anonymous, with no incentives provided. Before accessing the questionnaire, a brief introduction was displayed, and participants reviewed an electronic consent statement and confirmed their willingness to participate by selecting “Yes” to a consent question; only those who consented could proceed.

Data collection continued until the required sample size was reached, or no new responses were received for 48 hours. The final dataset included only participants who met the predefined inclusion criteria, ensuring representativeness of the adult population in Jazan.

### Statistical Analysis

The data were collected, reviewed, and then entered into SPSS version 26 (released 2019; IBM Corp). All statistical methods used were 2-tailed with an α level of .05, and results were considered significant if *P*≤.05. An overall knowledge score was computed by summing the correct answers, where a correct answer was given a 1-point score and 0 was given otherwise. Participants with a knowledge score less than 60% of the total correct answers were classified as having poor knowledge level, while others with a knowledge score of 60%-100% were classified as having good knowledge level. 

Descriptive analysis for categorical data was done using frequencies and percentages, whereas numerical data were presented as mean with standard deviation. Moreover, participants' knowledge and awareness about burn causes and first aid management, practice, history of burn, and post-burn medical consultation, management, complications, and daily effects were tabulated, while the overall knowledge level, source of information, preventive measures, and the available traditional treatments were graphed. We conducted cross-tabulations to examine factors associated with participants’ knowledge by using the Pearson chi-square test, and exact tests were applied for small cell counts.

### Ethical Considerations

This study followed the Declaration of Helsinki and Saudi national ethics guidelines, with approval from Jazan University’s Research Ethics Committee (REC-45/09/1030). It involved minimal risk human research. Participants aged 18 years or older from the Jazan region were recruited via an electronic self-administered questionnaire after providing electronic informed consent. Data were collected anonymously, with no personal identifiers, and stored securely on password-protected servers accessible only to the research team. Participation was voluntary, with no compensation provided, and no identifiable data or images were collected or published.

## Results

A total of 404 eligible participants completed the study questionnaire. The mean age was 26.8 (SD 11.9) years (range 18 to >60 years). Among them, 67.3% (272/404) were females, 72.8% (294/404) held a bachelor’s degree, and 96.8% (391/404) were Saudi nationals. Regarding employment status, 46.8% (189/404) were students and 30.7% (124/404) were employed. Monthly income was less than US $1332.50 for 28.2% (114/404) of the participants, between US $1332.50 and US $2665 for 29.5% (119/404) of the participants, and between US $2665 and US $5330.10 for 31.7% (128/404) of the participants. Concerning housing type, 41.8% (169/404) of the participants lived in apartments, 39.1% (158/404) in villas, and 19.1% (77/404) in traditional houses. Most participants (321/404, 79.5%) reported having children or teenagers in their homes. In terms of social media use, 65.6% (265/404) used Twitter, with 47.8% (193/404) of them for less than 2 hours daily. Facebook was used by 18.3% (74/404) of the participants, 15.6% (63/404) of whom used it for less than 2 hours daily. YouTube was used by 87.6% (354/404) of the participants, 56.2% (227/404) of whom reported daily use of less than 2 hours (Table 1).

**Table 1:** Sociodemographic characteristics and social media use among study participants in Jazan, Saudi Arabia (N=404).

Category		Values, n (%)
**Age (y)**		
	18-30	279 (69.1)
	31-45	68 (16.8)
	46-60	52 (12.9)
	>60	5 (1.2)
**Sex**		
	Male	132 (32.7)
	Female	272 (67.3)
**Educational level**		
	Secondary/below	54 (13.4)
	Diploma/institute	30 (7.4)
	Bachelor's degree	294 (72.8)
	Postgraduate	26 (6.4)
**Nationality**		
	Non-Saudi	13 (3.2)
	Saudi	391 (96.8)
**Employment**		
	Not working	91 (22.5)
	Student	189 (46.8)
	Working	124 (30.7)
**Monthly income**		
	<US $1332.50	114 (28.2)
	US $1332.5 - US $2665	119 (29.5)
	US $2665 - US $5330.10	128 (31.7)
	>US $5330.10	43 (10.6)
**Type of accommodation**		
	Popular house	77 (19.1)
	Apartment	169 (41.8)
	Villa	158 (39.1)
**Children or teenagers in home**		
	Yes	321 (79.5)
	No	83 (20.5)
**Daily Twitter use (h)**		
	Never use	135 (33.4)
	<2	193 (47.8)
	2-4	52 (12.9)
	>4	24 (5.9)
**Daily Facebook use (h)**		
	Never use	330 (81.7)
	<2	63 (15.6)
	2-4	5 (1.2)
	>4	6 (1.5)
**Daily YouTube use (h)**		
	Never use	50 (12.4)
	<2	227 (56.2)
	2-4	86 (21.3)
	>4	41 (10.1)

Table 2 shows the participants’ knowledge (N=404) and awareness regarding burn causes and first aid management in the Jazan region. More than half of the participants (236/404, 58.4%) had previously received information about burn prevention. For first-degree burns, 206 (51%) correctly identified washing the affected area with cold water as the appropriate first-aid measure, whereas 99 (24.5%) believed that applying ointments or creams was suitable. For second-degree burns, 154 (38.1%) correctly selected visiting the nearest health facility, while 152 (37.6%) preferred using ointments. Knowledge improved with burn severity, as 295 (73%) participants recognized that third-degree burns require urgent hospital care. A smaller proportion selected incorrect options, including surgical intervention (19/404, 4.7%) and water application (9/404, 2.2%).

For fourth-degree burns, 203 (50.2%) correctly selected emergency treatment, 113 (28%) preferred surgical intervention, and 71 (17.6%) were uncertain. Regarding specific first-aid practices (see Multimedia Appendix 2), 226 (55.9%) agreed that burns should be cooled under running water, and 220 (54.5%) disagreed with applying ice. In addition, 213 (52.7%) agreed that sterile gauze should be used to cover the affected area, and 284 (70.3%) recognized the importance of keeping the area ventilated. Awareness of conditions requiring urgent medical care was high: 276 (68.3%) for burns on joints, 303 (75%) for patients younger than 4 years or older than 70 years, and 350 (86.6%) for chemical or electrical burns. Attitudes toward reconstructive management were positive. A large proportion (353/404, 87.4%) acknowledged the role of plastic surgeons, and 260 (64.4%) participants considered plastic surgery safe and effective for restoring function and appearance. Overall, 228 (56.4%) participants had poor knowledge and 176 (43.6%) had good knowledge. The internet was the most common source of information (171/236, 72.5%), followed by formal courses (76/236, 32.2%), printed leaflets (75/236, 31.8%), television (67/236, 28.4%), newspapers (23/236, 9.7%), and radio (14/236, 5.9%) (Table 2).

Table 3 summarizes the history of burn incidents and reported first aid practices among participants in the Jazan region (N=404). A total of 215 (53.2%) participants reported that they or a close contact had experienced a burn injury. Among these, hot water (136/215, 63.8%) was the most frequent cause, followed by fire (105/215, 48.8%), chemical exposure (13/215, 6.1%), electrocution (4/215, 1.9%), and sunburn (2/215, 0.9%). The most commonly affected sites were the hands (111/215, 51.6%) and arms (85/215, 39.5%), followed by the thighs (49/215, 22.8%), chest (43/215, 20.3%), feet (35/215, 16.3%), back or buttocks (29/215, 13.5%), head (12/215, 5.6%), abdomen (3/215, 1.4%), and face (2/215, 0.9%). Regarding burn severity (n=215), 89 (41.4%) had first-degree burns, 70 (32.6%) had second-degree burns, 26 (12.1%) had third-degree burns, and 30 (14%) had fourth-degree burns. After the burn incident, 171 (79.5%) removed clothing and accessories from the affected area, and 129 (60%) sought medical assistance; 93 (43.3%) participants covered the injured site with a clean cloth, and 144 (66.9%) poured water on the burn, 114 (79.2%) of whom used cold water. Among those who used cold water (n=144), 62 (43.1%) applied it for 1-5 minutes, 39 (27.1%) for <1 minute, 21 (14.6%) for 5-10 minutes, 13 (9%) for 10-15 minutes, 3 (2.1%) for 15-20 minutes, and 6 (4.2%) for >20 minutes (Table 3).

**Table 2:** Knowledge and awareness of burn causes and first aid management in Jazan region (N=404).

Items		Values, n (%)
**Received information about burn prevention methods**		
	Yes	236 (58.4)
	No	168 (41.6)
**First aid for first-degree burns (superficial)**		
	Wash with cool water	206 (51)
	Apply ointments/creams	99 (24.5)
	Go to emergency unit	52 (12.9)
	I do not know	47 (11.6)
**First aid for second-degree burns (partial thickness)**		
	Go to emergency unit	154 (38.1)
	Apply ointments/creams	152 (37.6)
	Wash with cool water	38 (9.4)
	I do not know	60 (14.9)
**First aid for third-degree burns (deep partial thickness)**		
	Go to emergency unit	295 (73)
	Apply ointments/creams	20 (5)
	Surgical cosmetic intervention	19 (4.7)
	Wash with cool water	9 (2.2)
	I do not know	61 (15.1)
**First aid for fourth-degree burns (full thickness)**		
	Go to emergency unit	203 (50.2)
	Surgical cosmetic intervention	113 (28)
	Wash with cool water	12 (3)
	Apply ointments/creams	5 (1.2)
	I do not know	71 (17.6)
**Overall knowledge level**		
	Poor	228 (56.4)
	Good	176 (43.6)
**Source of information (n=236)**		
	Internet	171 (72.5)
	Formal course	76 (32.2)
	Paper leaflets	75 (31.8)
	Television	67 (28.4)
	Daily newspapers	23 (9.7)
	Radio	14 (5.9)

**Table 3:** History of burns and reported practice toward burn causes and first aid management in Jazan region.

Variable		Values, n (%)
**Have you or someone close to you been burned before? (N=404)**		
	Yes	215 (53.2)
	No	189 (46.8)
**Cause of burn (n=215)**		
	Hot water	136 (63.2)
	Fire	105 (48.8)
	Chemical materials	13 (6)
	Electrocution	4 (1.9)
	Sunburns	2 (0.9)
**Site of burn (n=215)**		
	Hand	111 (51.6)
	Arm	85 (39.5)
	Thigh	49 (22.8)
	Chest	43 (20)
	Foot	35 (16.3)
	Back and buttocks	29 (13.5)
	Head	12 (5.6)
	Abdomen	3 (1.4)
	Face	2 (0.9)
**Degree of burn (n=215)**		
	First degree	89 (41.4)
	Second degree	70 (32.6)
	Third degree	26 (12.1)
	Fourth degree	30 (14)
**Removed clothing/accessories after burn (n=215)**		
	Yes	171 (79.5)
	No	44 (20.5)
**Sought or arranged first medical assistance (n=215)**		
	Yes	129 (60)
	No	86 (40)
**Covered injury site with clean cloth (n=215)**		
	Yes	93 (43.3)
	No	122 (56.7)
**Poured water on burn area (n=215)**		
	Yes	144 (66.9)
	No	69 (32.1)
**Was the water cold? (n=144)**		
	Yes	114 (79.2)
	No	30 (20.8)
**Duration of water pouring (n=144)**		
	Less than a minute	39 (27.1)
	1-5 min	62 (43.1)
	5-10 min	21 (14.6)
	10-15 min	13 (9)
	15-20 min	3 (2.1)
	>20 min	6 (4.2)

Table 4 presents findings regarding post–burn medical consultation, management, complications, and the effect on daily activities among participants with a history of burn injury (n=215). Following a burn injury, 123 (57.2%) participants sought care at a health or emergency unit, while 92 (42.8%) did not. Among those who received medical evaluation (n=123), 118 (95.9%) were diagnosed and treated by a physician. The most commonly prescribed treatments were topical creams or ointments (87/118, 73.7%), bandages (24/118, 20.3%), surgical procedures (6/118, 5.1%), and medications (1/118, 0.8%). After treatment, 93.4% (110/118) reported clinical improvement, while 5.6% (8/118) did not. Complications were noted in 38 (17.7%) of the 215 participants, primarily complete deformation of skin appearance (28/38, 74%), followed by large blisters (4/38, 11%), scars (3/38, 8%), bacterial infection (1/38, 3%), bone or joint problems (1/38, 3%), and loss of affected parts (1/38, 3%). In addition, of the 215 participants, 36 (16.7%) participants indicated that burn injuries interfered with daily activities, while 179 (83.3%) stated that it did not (Table 4).

**Table 4:** Post–burn medical consultation, management, complications, and daily effect.

Variable			Values, n (%)
**Went to health/emergency unit after burn (n=215)**			
	Yes	123 (57.2)	
	No	92 (42.8)	
**Diagnosed and prescribed treatment by a doctor (n=123)**			
	Yes	118 (95.9)	
	No	5 (4.1)	
**Type of prescribed intervention** **(n=118)**			
	Creams or ointments	87 (73.7)	
	Bandages	24 (20.3)	
	Drugs	1 (0.8)	
	Surgical intervention	6 (5.1)	
**Condition improved after treatment** **(n=118)**			
	Yes	110 (93.4)	
	No	8 (6.8)	
**Complications occurred after the burn** **(n=215)**			
	Yes	38 (17.7)	
	No	177 (82.3)	
**Type of complications** **(n=38)**			
	Complete skin deformation	28 (73.7)	
	Bacterial infections	1 (2.6)	
	Bone and joint problems	1 (2.6)	
	Large blisters	4 (10.5)	
	Burn marks/scars	3 (7.9)	
	Loss of affected parts	1 (2.6)	
**Burn became an obstacle to daily activity** **(n=215)**			
	Yes	36 (16.7)	
	No	179 (83.3)	

Household safety measures were limited; 338 (83.7%) of the 404 participants preferred having a unified emergency number rather than multiple separate numbers. Only 103 (25.5%) had a fire extinguisher in their homes, 85 (21%) had an established evacuation plan, and 70 (17.3%) had practiced it. Smoke alarms were present in 33 (8.2%) homes, and 83 (20.5%) participants reported storing incendiary chemicals or flammable materials in a designated area.

Traditional remedies for burns were frequently reported (see Multimedia Appendix 3); 248 (61.4%) of the 404 participants reported using honey, followed by toothpaste (102/404, 25.2%), tomato paste (54/404, 13.4%), and aloe vera oil (16/404, 4%). Other less frequently reported remedies included flour (14/404, 3.5%), lavender oil (6/404, 1.5%), burn ointment (6/404, 1.5%), and mustard oil (5/404, 1.2%). Regarding the preferred methods for raising awareness about burn prevention and primary care, most participants favored visual educational materials (315/404, 78%) as the preferred medium for burn prevention awareness, followed by YouTube (229/404, 56.7%) and Twitter (195/404, 48.3%). Fewer respondents selected Facebook (70/404, 17.3%), Snapchat (65/404, 16.1%), newspapers (52/404, 12.9%), TikTok (21/404, 5.2%), and other sources (19/404, 4.7%).

Table 5 illustrates the factors associated with participants’ overall knowledge of burn causes and first aid management among the 404 respondents. Among participants aged 18-30 years (n=279), 159 (57%) had poor knowledge and 120 (43%) had good knowledge (*P*=.87). Sex was significantly associated with knowledge level (*P*=.043): females (272/404, 46.3% good knowledge) demonstrated higher knowledge than males (132/404, 37.9%). Educational level also showed a significant relationship (*P*=.049): postgraduates (15/26, 58%) exhibited the highest knowledge level, followed by secondary education or below (26/54, 48%). No significant associations were found for nationality (*P*=.18), employment status (*P*=.68), or having children or teenagers at home (*P*=.48). Participants who had previously received information about burn prevention (236/404, 50.8% good knowledge) had significantly higher scores than those who had not (*P*=.001). A prior history of burn injury (104/215, 48.4% good knowledge) was also significantly associated with better knowledge (*P*=.04). The source of information did not significantly affect knowledge levels (*P*=.47) (Table 5).

**Table 5:** Factors associated with participants’ knowledge of burn causes and first aid management.

Factor			Overall knowledge level, n (%)					*P* value^a^
			Poor		Good			
**Age (y)**								.87^b^
	18-30 (n=279)	159 (57)		120 (43)				
	31-45 (n=68)	37 (54.4)		31 (45.6)				
	46-60 (n=52)	30 (57.7)		22 (42.3)				
	>60 (n=5)	2 (40)		3 (60)				
**Sex**								.043^c^
	Male (n=132)	82 (62.1)		50 (37.9)				
	Female (n=272)	146 (53.7)		126 (46.3)				
**Educational level**								.049^c^
	Secondary/below (n=54)	28 (51.9)		26 (48.1)				
	Diploma/institute (n=30)	20 (66.7)		10 (33.3)				
	Bachelor's degree (n=294)	169 (57.5)		125 (42.5)				
	Postgraduate (n=26)	11 (42.3)		15 (57.7)				
**Nationality**								.18
	Saudi (n=391)	223 (57)		168 (43)				
	Non-Saudi (n=14)	5 (38.5)		8 (61.5)				
**Employment**								.68
	Not working (n=91)	55 (60.4)		36 (39.6)				
	Student (n=189)	105 (55.6)		84 (44.4)				
	Working (n=124)	68 (54.8)		56 (45.2)				
**Children or teenagers in home**								.48
	Yes (n=321)	184 (57.3)		137 (42.7)				
	No (n=83)	44 (53)		39 (47)				
**Received information about burn prevention**								.001^c^
	Yes (n=236)	116 (49.2)		120 (50.8)				
	No (n=168)	112 (66.7)		56 (33.3)				
**Source of burn prevention information**								.47^b^
	Internet (n=171)	88 (51.5)		83 (48.5)				
	Television (n=67)	33 (49.3)		34 (50.7)				
	Formal course (n=76)	32 (42.1)		44 (57.9)				
	Paper leaflets (n=75)	34 (45.3)		41 (54.7)				
	Daily newspapers (n=23)	14 (60.9)		9 (39.1)				
	Radio (n=14)	7 (50)		7 (50)				
**Personal or close burn experience**								.04^c^
	Yes (n=215)	111 (51.6)		104 (48.4)				
	No (n=189)	117 (61.9)		72 (38.1)				

^a^Pearson *X*^2^ test.

^b^Exact probability test.

^c^Significant at *P*<.05.

## Discussion

This study aimed to assess public knowledge and practice toward burns first aid management. In 2010, the results of 71 studies were analyzed in a systematic review of burn injuries that covered 12 of the 22 countries that make up the East Mediterranean region [19]. Overall, the incidence of burn injuries varied between 112 and 518 per 100,000 people. Nine of these 71 studies came from Saudi Arabia. However, there is no published summary of burn injuries in Saudi Arabia up to this point. There have been several concerns in the past few years to lower the risk of burns by raising public awareness and motivating people to take burn prevention steps. Consequently, there appears to have been a decline in burn incidence and burn-related mortality, particularly in high-income nations [19].

As for participants’ personal or family history of burns, our study reveals that 53.2% (215/404) of the total participants had experienced burns before—with hot water, fire, and chemicals being the most common causes. The most common burn sites were the hands, arm, and thigh. Most participants had first-degree burns, followed by medical assistance and management received by more than half of the respondents with burns. Most went to the health/emergency unit, with most receiving treatment from doctors. Most treated patients showed an improvement, but 17.7% (38/215) experienced complications, including skin deformation and difficulty in daily activities. A previous study in Jazan by Hoque et al [20] revealed that nearly three-fourths of the participants had experienced a burn themselves or in a family member, which is higher than the assessed prevalence among this study’s respondents. Thermal burns were the most dominant similar to that reported in this study.

Considering knowledge and awareness, this study revealed that more than half of the participants received information about burn prevention methods mainly from the internet and formal courses. Study participants showed high awareness regarding the importance of medical consultation, visiting emergency for burns and the cornerstone role of plastic surgeons in burns. Knowledge of the initial first aid steps for burns was substantially lower. Generally, less than half of the study participants were knowledgeable about burns causes and first aid management. Similar findings in Jazan region were reported by Hoque et al [20], as 69.9% of the participants had inadequate knowledge, although 72% of participants had a favorable attitude toward burn first aid. Additionally, a Jordanian study found that 59% of the participants knew that covering the burned area with a clean cloth can reduce the risk of infection [21]. However, the Jordanian study showed a lower percentage of participants knowing about covering the burned area correctly with a clean cloth. That study also found that a significant proportion of participants believed that toothpaste could be used to treat burns, which is a common misconception. A much better knowledge was reported by Abu et al [22], the most recent study in Jazan, where 91.8% (n=367) knew to use water, 50.8% (n=203) would seek medical attention, 22.6% (n=90) would cover burns, and 20.3% (n=81) considered pain management important. This suggests that adults in Jazan have a good understanding of the causes, prevention, and first aid procedures for burn injuries in contrast to our study findings.

Although a substantial proportion of participants reported using the internet as a primary source of health information, the overall knowledge about burns first aid remained low. This discrepancy may be explained by the quality and reliability of online information accessed by the general public. Many people rely on social media and other informal online sources, where misinformation is widespread and scientific accuracy is often missing. In addition, most individuals in the region participate with health information passively, accessing it by chance rather than actively searching for reliable, evidence-based guidance on emergency care such as burn first aid.

Cultural beliefs and traditional practices also play a significant role in shaping burn management behaviors. The use of home remedies such as honey, toothpaste, tomato paste, or aloe vera oil means long-standing community traditions and trust in folk medicine passes down through generations. These practices are often reinforced through family, friends, and social networks, sometimes even shared on digital platforms, further perpetuating misconceptions. In addition, limited exposure to official first aid training programs and a general perception that slight burns can be self-treated at home contribute to inappropriate first aid responses.

Regarding post-burn practice, our study shows that more than half of the patients with burn injuries went to the health/emergency unit, with 95.9% (118/123) diagnosed and prescribed treatment. Most patients received creams or ointments and bandages, with very few cases requiring surgery. Most patients experienced an improvement, with complications in less than one-fifth of the cases involving skin deformation and 16.7% (36/215) affecting daily activities. A large-scale study in the western region of Saudi Arabia revealed that most patients with burn injuries (84.2%) had treated their burns with traditional medication [23]. Because most respondents agreed incorrectly that antibiotics are helpful in cases of burns, there was a lack of knowledge regarding the use of antibiotics in burn injuries.

Honey is thought to be a potentially promising therapy because it can promote tissue growth, increase epithelialization, and decrease scar formation [24]. As shown in our study, about two-thirds of the participants selected honey as a first-aid burn treatment as the most used conventional method. According to a Saudi Arabian study, 69.9% of the participants use honey as a burn treatment in concordance to our study finding [25]. It should be highlighted that, when compared to early excision and grafting, the results of a systematic review that compared the use of honey to other conventional dressing procedures revealed significant uncertainties and may postpone the ability of partial and full-thickness burns to heal [26,27].

In conclusion, our study reveals unsatisfactory knowledge and awareness about burn causes and first aid management among the Jazan public. Higher education, female sex, and a personal or family history of burns were associated with higher knowledge levels. Internet was the most commonly reported source of information, with seeking medical consultation among nearly half of the cases. Nearly half of the participants had experienced burns or known someone had, mainly on the hands, arms, and thigh. Thermal burns were the most frequent. The findings suggest a lack of awareness among the general population about the appropriate first-aid measures for burns, and health care professionals should take an active role in educating patients and communities about burn first aids.

## Data Availability

Data may be available upon request to the corresponding author.

## Appendix

Multimedia Appendix 1 Structured questionnaire administered in Arabic.

Multimedia Appendix 2 Burns first-aid practices.

Multimedia Appendix 3 Traditional remedies for burns.

## Abbreviations

LMICs: low- and middle-income countries
WHO: World Health Organization
