# Supplementary material for: Knowledge, Attitudes, and Practices Toward Burn Causes and First Aid Management in Jazan Region, Saudi Arabia: Cross-Sectional Study
Source: JMIR Form Res. 2025 Dec 23;9:e80594. doi: 10.2196/80594 (PMC12775758; doi:10.2196/80594)
Supplement: Multimedia Appendix 1 [file formative_v9i1e80594_app1.docx]

**Appendix 1:** Questionnaire

*This questionnaire assesses public awareness of burn causes and first-aid management in the Jazan region. All responses are confidential and will be used solely for scientific research. By participating, you contribute valuable insights to strengthen community awareness and first-aid practices for burns.*

**Consent**

By checking the option below, you confirm that you have read the study information and agree to participate.

○ Do you agree to participate in this study? *

- Yes
- No

**Section A - Demographics**

1) What is your age group? *

○ Under 18 years

○ 18–30 years

○ 31–45 years

○ 46–60 years

○ Over 60 years

2) What is your gender? *

○ Male

○ Female

3) What is your highest level of education? *

○ No formal education

○ Primary

○ Intermediate

○ Secondary

○ Institute / Diploma

○ Bachelor’s

○ Postgraduate

4) What is your nationality? *

○ Saudi

○ Non-Saudi

5) Where is your current residence? *

| ○ Jazan | ○ Abu Arish |
| --- | --- |
| ○ Samtah | ○ Sabya |
| ○ Baish | ○ Other: ______ |

6) Employment status *

○ Employed

○ Unemployed

○ Student

7) What is your household’s total monthly income? *

○ Less than 5,000 SAR

○ 5,000–10,000 SAR

○ 10,000–20,000 SAR

○ More than 20,000 SAR

8) What type of housing do you live in? *

○ Apartment

○ Villa

○ Traditional house

9) Are there children or adolescents (under 18 years) living in your home? *

○ Yes

○ No

**Section B - Prior Information & Sources**

10) Have you previously received information about burn prevention? *

○ Yes

○ No (skip to Q12)

11) If yes, where did you receive information about burn prevention? (Select all that apply)

☐ Official course

☐ Printed brochures/leaflets

☐ Newspapers

☐ Television

☐ Radio

☐ Internet

12) Have you previously received information about first aid for burns? *

○ Yes

○ No (skip to Q14)

13) If yes, where did you receive information about first-aid for burns? (Select all that apply)

☐ Official course

☐ Printed brochures/leaflets

☐ Newspapers

☐ Television

☐ Radio

☐ Internet

**Section C - First Aid Knowledge**

Note on burn depth (WHO, simplified): First-degree = superficial (red, painful, no blisters); Second-degree = partial-thickness (blisters, wet/pink); Third-degree = full-thickness (white/charred, may be less painful); Fourth-degree = extends into deeper tissues. This note is for clarity only.

14) What is the appropriate first aid for a first-degree (superficial) burn? *

○ Rinse the injured area under cool running water

○ Apply ointments/medical creams

○ Go to the nearest health facility / Emergency department

○ Cosmetic surgical intervention

○ I do not know

15) What is the appropriate first aid for a second-degree (partial-thickness) burn? *

○ Rinse the injured area under cool running water

○ Apply ointments/medical creams

○ Go to the nearest health facility / Emergency department

○ Cosmetic surgical intervention

○ I do not know

16) What is the appropriate first aid for a third-degree (full-thickness) burn? *

○ Rinse the injured area under cool running water

○ Apply ointments/medical creams

○ Go to the nearest health facility / Emergency department

○ Cosmetic surgical intervention

○ I do not know

17) What is the appropriate first aid for a fourth-degree burn (involving deeper tissues)? *

○ Rinse the injured area under cool running water

○ Apply ointments/medical creams

○ Go to the nearest health facility / Emergency department

○ Cosmetic surgical intervention

○ I do not know

**Section D - Attitudes Toward Burn First Aid**

18) Please choose one response for each statement: *

| Statement | Agree | Disagree | Don’t know |
| --- | --- | --- | --- |
| Burned clothing stuck to the skin should be removed. | ○ | ○ | ○ |
| Cool running water should be poured on the burn area. | ○ | ○ | ○ |
| Ice should be applied directly to the burn area. | ○ | ○ | ○ |
| After removing loose clothing, the burn should be covered with sterile gauze. | ○ | ○ | ○ |
| The burn area should be left uncovered to ventilate. | ○ | ○ | ○ |
| Going to the ER depends on the body area affected. | ○ | ○ | ○ |
| You must go to the ER if the patient is younger than 4 or older than 70. | ○ | ○ | ○ |
| You must go to the ER if the burn is over a joint. | ○ | ○ | ○ |
| You must go to the ER for chemical or electrical burns. | ○ | ○ | ○ |
| A plastic surgeon may intervene if there are scars/deformities after the burn. | ○ | ○ | ○ |
| Cosmetic surgery after burns is safe and effective. | ○ | ○ | ○ |

**Section E - Personal or Family Burn Experience**

19) Have you (or a close relative) experienced a burn before? *

○ Yes

○ No (skip to Q36)

20) Where on the body was the burn? (Select all that apply)

☐ Head

☐ Chest

☐ Arm

☐ Hand

☐ Back & buttocks

☐ Leg

☐ Foot

☐ Other: _____

21) What was the cause of the burn? (Select all that apply)

☐ Sun exposure

☐ Fire/flame

☐ Electric shock

☐ Hot liquids (e.g., boiling water/oil)

☐ Chemicals

☐ Other: ______

22) What was the degree of the burn?

○ First-degree

○ Second-degree

○ Third-degree

○ Fourth-degree

○ I do not know

23) After the burn, did you remove clothing and accessories (e.g., watches, jewelry) from the injured area?

○ Yes

○ No

24) After the burn, did you seek or coordinate initial medical help?

○ Yes

○ No

25) After the burn, did you cover the area with a clean cloth after removing surrounding clothing?

○ Yes

○ No

26) Did you pour water over the burn area?

○ Yes

○ No

27) If yes, was the water cool?

○ Yes

○ No

28) If you poured water over the burn area, for how long?

○ Less than 1 minute

○ 1–5 minutes

○ 5–10 minutes

○ 10–15 minutes

○ 15–20 minutes

○ More than 20 minutes

29) After the burn, did you go to a health facility / emergency department?

○ Yes

○ No

30) Were you diagnosed by a physician and given treatment?

○ Yes

○ No

31) What treatment did you receive?

○ Dressings

○ Creams or ointments

○ Surgery

○ Oral medications

○ Other: ______

32) Did your condition improve after treatment?

○ Yes

○ No

33) Did any complications occur after the burn?

○ Yes

○ No

34) If yes, which of the following occurred? (Choose one)

☐ Complete deformity of the skin at the burn site

☐ Loss of body parts at the burn site

☐ Bacterial infection of the burn area

☐ Large blisters

☐ Severe drop in body temperature (hypothermia)

☐ Difficulty breathing

☐ Bone or joint problems

☐ Other: ______

35) Did the burn become an obstacle to practicing daily activities?

○ Yes

○ No

**Section F - Scenarios & Household Safety**

36) Which traditional remedies are commonly used to treat burns in your community, if any? (Select all that apply)

☐ Honey

☐ Toothpaste

☐ Tomato paste

☐ A piece of fruit

☐ Lavender oil

☐ Aloe vera

☐ Mustard oil

☐ Soy oil

☐ None

☐ Other: ______

37) Hot oil spills on a child’s chest at home. What is the best immediate action? *

○ Remove the shirt and place the burn under cool running water for 15 minutes

○ Remove the shirt and apply ice cubes to the burn

○ Keep the clothes on and call for help

○ Keep the clothes on and pour ice water on the burn

○ I don’t know

38) A person’s clothes catch fire during an outdoor picnic. What action is most appropriate? *

○ Find water and spray it on the person

○ Tell them to stop, drop, and roll

○ Tell them to remove their clothes and apply ice to the burn area

○ I don’t know

39) Boiling water spills on a person’s hand at a social gathering. What is the best first step? *

○ Apply ice cubes to the burn

○ Place the burn under cool water for 10 minutes

○ Cover the burn with a clean cloth and call for help

○ I don’t know

40) Do you have the following burn-prevention items at home? (Yes/No for each)

| Item | Response |
| --- | --- |
| Smoke alarm | ○ Yes ○ No |
| Fire extinguisher | ○ Yes ○ No |
| Dedicated storage for corrosive/flammable chemicals | ○ Yes ○ No |
| Home evacuation plan | ○ Yes ○ No |

41) Do you think a single unified emergency number is better than separate numbers for each service? *

○ Yes

○ No

42) Have you practiced a home evacuation plan? *

○ Yes

○ No

43) In your opinion, which of the following are the best channels for awareness about burn prevention and first aid? (Select all that apply)

☐ Visual media (e.g., TV/videos)

☐ Radio

☐ Newspapers

☐ Twitter (X)

☐ YouTube

☐ Facebook

☐ Other: ______

44) How many hours do you spend daily on Twitter (X)?

○ Less than 2 hours

○ 2–4 hours

○ More than 4 hours

○ I don’t use Twitter (X)

45) How many hours do you spend daily on Facebook?

○ Less than 2 hours

○ 2–4 hours

○ More than 4 hours

○ I don’t use Facebook

46) How many hours do you spend daily on YouTube?

○ Less than 2 hours

○ 2–4 hours

○ More than 4 hours

○ I don’t use YouTube

Optional) If you would like to receive the study findings, please provide your email: ____________
