# Supplementary material for: Knowledge, Attitudes, and Practices Toward Burn Causes and First Aid Management in Jazan Region, Saudi Arabia: Cross-Sectional Study
Source: JMIR Form Res. 2025 Dec 23;9:e80594. doi: 10.2196/80594 (PMC12775758; doi:10.2196/80594)
Supplement: Multimedia Appendix 2 [file formative_v9i1e80594_app2.pdf]

Appendix 2: Attitude Towards Burn First Aid in the Jazan Region

Items	No (%)
**Burnt clothing stuck to skin must be removed**	
Agree	198 (49.0)
Disagree	143 (35.4)
I don't know	63 (15.6)
**Pour cold water on affected area**	
Agree	226 (55.9)
Disagree	117 (29.0)
I don't know	61 (15.1)
**Apply ice to affected area**	
Agree	98 (24.3)
Disagree	220 (54.5)
I don't know	86 (21.3)
**Cover affected area with sterile gauze after removing clothing**	
Agree	213 (52.7)
Disagree	114 (28.2)
I don't know	77 (19.1)
**Ventilate affected area**	
Agree	284 (70.3)
Disagree	59 (14.6)
I don't know	61 (15.1)
**Emergency visit depends on affected area**	
Agree	246 (60.9)
Disagree	117 (29.0)
I don't know	41 (10.1)
**Emergency visit if patient <4 or >70 years old**	
Agree	303 (75.0)
Disagree	63 (15.6)
I don't know	38 (9.4)
**Emergency visit if burn is on a joint**	
Agree	276 (68.3)
Disagree	39 (9.7)
I don't know	89 (22.0)
**Emergency visit for chemical/electrical burns**	
Agree	350 (86.6)
Disagree	19 (4.7)
I don't know	35 (8.7)
**Plastic surgeon can intervene for burn deformities**	
Agree	353 (87.4)
Disagree	12 (3.0)
I don't know	39 (9.7)
**Plastic surgery after burn is safe and effective**	
Agree	260 (64.4)
Disagree	27 (6.7)
I don't know	117 (29.0)
