# Supplementary material for: Knowledge, Attitudes, and Practices Toward Burn Causes and First Aid Management in Jazan Region, Saudi Arabia: Cross-Sectional Study
Source: JMIR Form Res. 2025 Dec 23;9:e80594. doi: 10.2196/80594 (PMC12775758; doi:10.2196/80594)
Supplement: Multimedia Appendix 3 [file formative_v9i1e80594_app3.pdf]

Appendix 3: demonstrates the availability of traditional remedies for burns among participants in the Jazan Region.

**Treatments**	**No. (%)**
Honey	248 (61.4%)
Toothpaste	102 (25.2%)
None	78 (19.3%)
Tomato paste	54 (13.4%)
Others	25 (6.2%)
Aloe vera oil	16 (4.0%)
Flour	14 (3.5%)
Burn ointment	6 (1.5%)
Lavender oil	6 (1.5%)
Mustard oil	5 (1.2%)
